# Coupled harmonic oscillators model with two connected point masses for application in photo-induced force microscopy

**DOI:** 10.1515/nanoph-2023-0424

**Published:** 2023-09-07

**Authors:** Junghoon Jahng, Eun Seong Lee

**Affiliations:** Hyperspectral Nanoimaging Team, Korea Research Institute of Standards and Science (KRISS), Daejeon 34113, South Korea

**Keywords:** Photo-Induced Force Microscopy, coupled harmonic oscillators, thermal expansion force

## Abstract

To comprehensively describe the operation of photo-induced force microscopy (PiFM), we have developed a model based on coupled harmonic oscillators. This model features two point masses connected by massless elastic wires, offering greater intuitiveness compared to existing PiFM models. It simplifies these models into a unified theoretical framework. By solving the equations of motion using adjusted oscillator parameters, we have successfully replicated all dynamic features from previous theories. These features include resonance frequencies and shapes of eigenmodes, as well as the responses to various external forces in the two PiFM modes: direct coupling and sideband coupling. Furthermore, by integrating our model with a recently developed photo-induced thermal expansion force model, which covers both tip-enhanced and global expansions, we have managed to uncover the underlying physical mechanism responsible for the unique signal behaviors observed in sideband coupling mode, where the signal plot, as a function of sample thickness, unexpectedly exhibits a peak followed by a valley, rather than a proportionally increasing signal. Our study has the potential to enhance the comprehension of various other physical phenomena associated with PiFM in the future.

## 1 Introduction

Since the invention of the atomic force microscope (AFM), numerous variants have emerged alongside the development of diverse probe fabrication techniques. These variations aim to map a wide range of material properties, including mechanical, electrical, magnetic, and thermal attributes, in addition to topography [1–8]. These properties are probed by examining various interactions between the tip and the sample through the motion of a cantilever. The introduction of the Multi-frequency atomic force microscope (MF-AFM) has enabled the encoding and decoding of a plethora of information about sample properties by analyzing the frequency components of the probe’s motion [9]. An exemplary instance is the bimodal AFM technique, where the first cantilever mode captures topography, while the second mode measures magnetic or electrical characteristics of the sample surface [10–13]. The successful applications of MF-AFM have rendered it a promising approach in the realm of Scanning Probe Microscopy (SPM).

A relatively recent advancement in this field is photo-induced force microscopy (PiFM) [14–18]. In PiFM, one of the cantilever’s multiple eigenmodes manages the gap between the tip and the sample, facilitating topography measurement. Meanwhile, another mode is dedicated to measuring photo-induced forces, allowing the extraction of optical spectroscopic information from the sample. Notably, PiFM offers simplicity compared to conventional nano-optical imaging methods like scanning near-field optical microscopy (SNOM) [19–21]. The absence of a need for a photodetector to measure scattered light alleviates concerns about signal reduction due to light loss, thereby ensuring heightened sensitivity. The optical response is exclusively reliant on the probing tip, enabling a wide measurement bandwidth spanning from ultraviolet to terahertz.

Initially, PiFM operated in the direct mode [15, 22], where the external laser beam’s modulation frequency aligned with one cantilever eigenmode, while the other mode was designated for topography. Force signals were obtained via demodulation at the light modulation frequency. An alternative approach is the sideband coupling (SC) method [23, 24], involving the mixing of the laser modulation frequency with one eigenmode, and aligning the resultant mixing frequency with the other eigenmode. This scheme, sensitive to the force gradient on the tip, yields sharper images compared to the direct mode. Moreover, it eliminates noise stemming from background forces that are not related to the tip-sample interaction.

We have previously reported theoretical analysis of these two operation modes through two research papers, where only the two lowest eigenmodes of the Euler–Bernoulli beam model are considered [15, 23]. However, we realize that in many cases, instead of describing AFM characteristics in terms of beam dynamics, a point mass simple harmonic oscillator is very useful to understand the system and captures many of the key features of AFM behavior [25, 26]. Similarly, a coupled harmonic oscillator with two point masses can be more intuitive and useful in describing the PiFM system and grasping the physical meaning of characteristic dynamic features. In this paper, we introduce a coupled oscillator model with two point masses connected by two massless elastic wires to describe the PiFM signal behaviors in detail, and develop a comprehensive thermal expansion force model that exhibits a different tip-sample distance dependence between tip-enhanced expansion and global one. Then we combine the two models to elucidate the physical mechanism behind the peculiar peak-and-valley-shaped plot of PiFM signals as a function of sample thickness in the SC mode experiments, meaning that the signal does not increase continuously with sample thickness. The previous PiFM studies of thermal expansion forces with small oscillation amplitude could not successfully explain this behavior in thick sample region (>1 μm). Although currently qualitative, it is clearly demonstrated through this combined model that the underlying physical mechanism for this signal behavior is a tip-enhanced thermal expansion. We expect that the presented model will facilitate the understanding of other various physical phenomena involved in PiFM studies.

## 2 Theoretical models

### 2.1 A coupled harmonic oscillators model with two connected point masses

**Figure 1:**
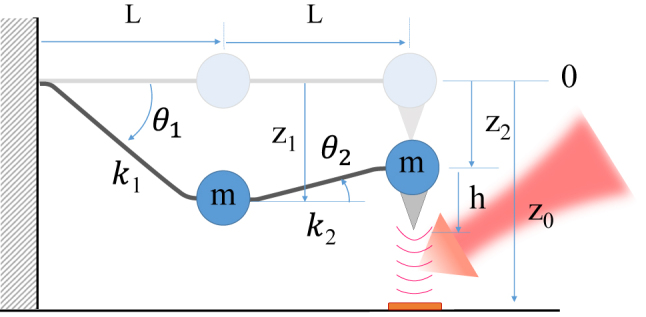
Coupled harmonic oscillators model having two point masses connected by two massless elastic wires to apply for PiFM. The tip-sample junction is illuminated by a laser beam to generate a force response that can characterize the optical properties of the sample.

To model the MF-AFM using a coupled harmonic oscillator comprised of point masses, it is necessary to connect a number of masses equal to the required eigenmodes with massless springs. Considering that PiFM is treated as a bimodal operation necessitating only two modes, describing it with two interconnected masses is adequate. To measure additional physical quantities, the number of point masses and springs can be augmented to accommodate the relevant modes. In this current study, we construct our model with two point masses connected in series by a massless elastic wire, as illustrated in Figure 1 below. A sharp tip is affixed to the end mass. Since these are point masses, no rotational motion is taken into consideration.

The equation of motion for *z*_1_ and *z*_2_ in this system can be expressed by:

(1) $$\text{m}{\ddot{z}}_{1}=-{\text{k}}_{1}{\text{z}}_{1}+{\text{k}}_{2}\left({\text{z}}_{2}-{\text{z}}_{1}\right)+{\text{F}}_{0}\text{cos}\omega_{\text{drv}}\text{t}$$

$$\text{m}{\ddot{z}}_{2}=-{\text{k}}_{2}\left({\text{z}}_{2}-{\text{z}}_{1}\right)+{\text{F}}_{\text{tip}}\left({\text{z}}_{0}-\text{h}-{\text{z}}_{2}\right)-\Gamma \left({\text{z}}_{0}-\text{h}\right){\dot{z}}_{2}$$

(2) $$\;\;\;\;\;\;\;\;\;\;\;\;\;\;\;\;+\;{\text{F}}_{\text{opt}}\left({\text{z}}_{0}-\text{h}-{\text{z}}_{2}\right)\text{co}{\text{s}}^{2}\left(\frac{\omega_{\text{opt}}}{2}\text{t}+\frac{\varphi }{2}\right)$$

where *m*, *k*_i_, and *z*_0_ denote the mass, *i*th spring constant, and the equilibrium position, respectively. The tip is oscillating under the influence of the conservative force field, *F*_tip_ (for example, Van der Waals interaction). The piezo-vibrator drives the system at the frequency *ω*_drv_ with a force strength *F*_0_, and the modulated laser beam illuminates the tip-sample junction to induce another driving force, the photo-induced force, *F*_opt_ at *ω*_opt_ and with phase *φ* relative to the piezo-vibrator oscillation. The coefficient Γ represents damping or dissipation, characterizing the non-conservative tip-sample interaction, wherein the resultant force is assumed to be proportional to the tip velocity. For simplicity, we neglect the gravitational force acting on the masses. We proceed to approximate the two forces *F*_tip_ and *F*_opt_ near the equilibrium position using the assumption of small displacements, *z*_*i*_.

(3) $${\text{F}}_{\text{opt}}\left({\text{z}}_{2}\right)\approx {\text{F}}_{\text{opt}}\left({\text{z}}_{0}\right)-g{\text{z}}_{2},{\text{F}}_{\text{tip}}\left({\text{z}}_{2}\right)\approx {\text{F}}_{\text{tip}}\left({\text{z}}_{0}\right)-K{\text{z}}_{2}$$

where *g* = d*F*_opt_/d*z* and *K* = d*F*_tip_/d*z* evaluated at *z* = *z*_0_. For the description of direct mode operation, we set *g* = 0 for now, and h is set to be zero for simplicity. We now can express the Eqs. (1) and (2) in a single vector format as

(4) $$\begin{array}{ll} & m\left(\begin{array}{c} {\ddot{z}}_{1}\\ {\ddot{z}}_{2} \end{array}\right)+\left(\begin{array}{cc} k_{1}+k_{2} & -k_{2}\\ -k_{2} & k_{2}+K \end{array}\right)\left(\begin{array}{c} {\text{z}}_{1}\\ {\text{z}}_{2} \end{array}\right)\\ & \;=\left(\begin{array}{c} {\text{F}}_{0}\text{cos}\omega_{\text{drv}}\text{t}\\ \frac{1}{2}{\text{F}}_{\text{opt}}\left({\text{z}}_{0}\right)\text{cos}\left(\omega_{\text{opt}}\text{t}+\varphi \right) \end{array}\right)-\Gamma \left(\begin{array}{c} 0\\ {\dot{z}}_{2} \end{array}\right) \end{array}$$

Diagonalizing the matrix yields a new equation expressed in terms of two new modes, *Q*_1_ and *Q*_2_, each with corresponding resonance frequencies of $\sqrt{\lambda_{1}/m}$ and $\sqrt{\lambda_{2}/m}$ , respectively. To account for internal damping, we introduce empirical dissipation terms *b*_1_ and *b*_2_.

(5) $$\begin{array}{ll} & m\left(\begin{array}{c} {\ddot{Q}}_{1}\\ {\ddot{Q}}_{2} \end{array}\right)+\left(\begin{array}{cc} b_{1}-U_{21}\Gamma & 0\\ 0 & b_{2}+U_{22}\Gamma \end{array}\right)\left(\begin{array}{c} {\dot{Q}}_{1}\\ {\dot{Q}}_{2} \end{array}\right)+\left(\begin{array}{cc} \lambda_{1} & 0\\ 0 & \lambda_{2} \end{array}\right)\\ & \;\times \left(\begin{array}{c} {\text{Q}}_{1}\\ {\text{Q}}_{2} \end{array}\right)={\tilde{U}}^{-1}\vec{F}\left(t\right)+\Gamma \left({\text{z}}_{0}\right)\left(\begin{array}{c} {\text{U}}_{22}{\dot{Q}}_{2}\\ -{\text{U}}_{21}{\dot{Q}}_{1} \end{array}\right) \end{array}$$

with $\vec{Q}={\tilde{U}}^{-1}\vec{Z}$ , $\tilde{U}=\left(\begin{array}{cc} \frac{1}{-2\sqrt{{\left({\text{y}}_{\text{K}}-{\text{y}}_{1}\right)}^{2}+1}} & \frac{1}{-2\sqrt{{\left({\text{y}}_{\text{K}}-{\text{y}}_{1}\right)}^{2}+1}}\\ \frac{y_{K}-{\text{y}}_{1}}{2\sqrt{{\left({\text{y}}_{\text{K}}-{\text{y}}_{1}\right)}^{2}+1}}-\frac{1}{2} & \frac{y_{K}-{\text{y}}_{1}}{2\sqrt{{\left({\text{y}}_{\text{K}}-{\text{y}}_{1}\right)}^{2}+1}}+\frac{1}{2} \end{array}\right),{\tilde{U}}^{-1}=\left(\begin{array}{cc} y_{1}-y_{K}-\sqrt{{\left(y_{K}-y_{1}\right)}^{2}+1} & -1\\ y_{K}-y_{1}-\sqrt{{\left(y_{K}-y_{1}\right)}^{2}+1} & 1 \end{array}\right)$ .

where $k_{2}/m=\omega_{R}^{2}$ , *k*_1_/*k*_2_ = 2*y*_1_, *K*/*k*_2_ = 2*y*_*K*_, $\lambda_{1}=m\omega_{R}^{2}$ [ $\left(y_{1}+y_{K}+1\right)-\sqrt{{\left(y_{K}-y_{1}\right)}^{2}+1}$ ], and $\lambda_{2}=m\omega_{\text{R}}^{2}\left[\left(y_{1}+y_{\text{K}}+1\right)\right.+\sqrt{{\left(y_{\text{K}}-y_{1}\right)}^{2}+1}$ ].

**Figure 2:**
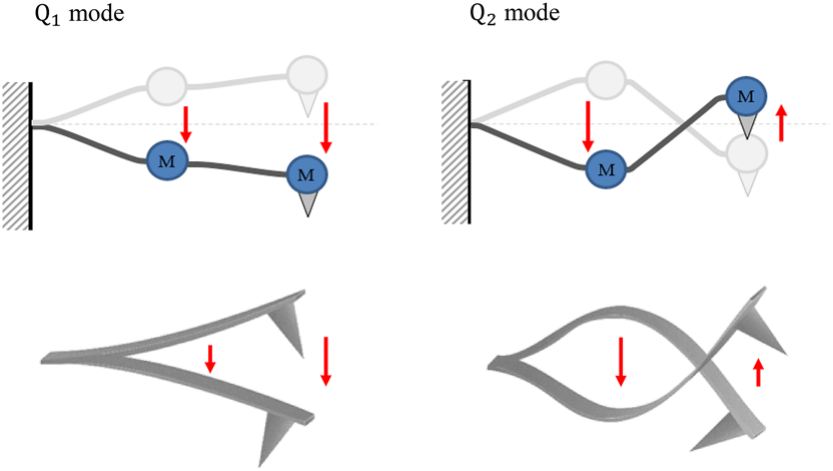
Motional shapes of the two eigenmodes of coupled harmonic oscillators. Below each mode is the corresponding beam-shaped cantilever eigenmode motion. It can be seen that the movement of the point masses is enough to represent the shape of the beam’s motion.

For a small value of Γ(z_0_), the case of the large tip-sample distance, we can ignore the last term and have two decoupled equations. The two modes *Q*_1_ and *Q*_2_ become eigenmodes of the system and can be resonantly excited by external harmonic driving frequencies, $\sqrt{\lambda_{1}/m}$ and $\sqrt{\lambda_{2}/m}$ , respectively. As the tip approaches to the sample surface, the Γ(*z*_0_) gets bigger. Even so, we still can ignore the term because we usually are driving the system near the resonance frequencies. The dissipative driving term $\Gamma {\dot{Q}}_{2}$ that oscillates as $\sqrt{\lambda_{2}/m}$ contributes little to the motion of the *Q*_1_ mode whose resonance frequency is very different from $\sqrt{\lambda_{2}/m}$ and vice versa. Now we have two decoupled harmonic oscillators with resonance frequencies of $\sqrt{\lambda_{1}/m}$ and $\sqrt{\lambda_{2}/m}$ as follows,

(6) $$\begin{array}{ll} {\ddot{Q}}_{1}+\gamma_{1}{\dot{Q}}_{1}+\frac{\lambda_{1}}{m}{\text{Q}}_{1} & =-\frac{0.95}{m}{\text{F}}_{0}\text{cos}\omega_{\text{drv}}t\\ & \;-\frac{1}{2m}{\text{F}}_{\text{opt}}\left({\text{z}}_{0}\right)\text{cos}\left(\omega_{\text{opt}}\text{t}+\varphi \right) \end{array}$$

(7) $$\begin{array}{ll} {\ddot{Q}}_{2}+\gamma_{2}{\dot{Q}}_{2}+\frac{\lambda_{2}}{m}{\text{Q}}_{2} & =-\frac{1.05}{m}{\text{F}}_{0}\text{cos}\omega_{\text{drv}}t\\ & \;+\frac{1}{2m}{\text{F}}_{\text{opt}}\left({\text{z}}_{0}\right)\text{cos}\left(\omega_{\text{opt}}\text{t}+\varphi \right) \end{array}$$

where *γ*_1_ = *b*_1_ − *U*_21_Γ and *γ*_2_ = *b*_2_ + *U*_22_Γ. Adjusting the parameters *m*, *k*_1_, and *k*_2_, we can make the eigenmode frequencies match the cantilever values used in experiments. From the fact that in typical cantilevers the second eigenmode frequency is 6.27 times higher than the first one, the *y*_1_ value is determined to be 0.05 as a good approximation. With an assumption *k*_1_ ≫ *K*, which is a typical case in AFM systems, it leads to the solutions of Eqs. (6) and (7) as below.

(8) $$\begin{array}{ll} Q_{1} & \approx -\frac{{\text{F}}_{\text{opt}}\left(z_{0}\right)}{2m\sqrt{{\left[\omega_{\text{opt}}^{2}-\omega_{01}^{2}-\frac{K}{2m}\right]}^{2}+\gamma_{1}^{2}\omega_{\text{opt}}^{2}}}\times \\ & \;\text{cos}\left(\omega_{\text{opt}}t+\Delta \right)\;\text{with}\;\omega_{01}^{2}=k_{1}/2m\;\text{and}\;▵\\ & =\varphi -\text{ta}{\text{n}}^{-1}\frac{\gamma_{1}\omega_{\text{opt}}}{\lambda_{1}/m-\omega_{\text{opt}}^{2}} \end{array}$$

and

(9) $$\begin{array}{ll} Q_{2} & \approx -\frac{1.05{\text{F}}_{0}}{m\sqrt{{\left[\omega_{\text{drv}}^{2}-\omega_{02}^{2}-\frac{K}{2m}\right]}^{2}+\gamma_{2}^{2}\omega_{\text{drv}}^{2}}}\times \\ & \;cos\left(\omega_{\text{drv}}t+\delta \right)\;\text{with}\;\omega_{02}^{2}=20k_{1}/m\;\text{and}\;\delta \\ & =\text{ta}{\text{n}}^{-1}\frac{\gamma_{2}\omega_{\text{drv}}}{\omega_{\text{drv}}^{2}-\lambda_{2}/m} \end{array}$$

where *ω*_01_ and *ω*_02_ are the eigenmode frequencies when the tip is very far from the sample surface where *K* = 0. Here, by setting the piezo-vibrator frequency *ω*_drv_ around $\omega_{2}\left(=\sqrt{\omega_{02}^{2}+K/2m}\right)$ and the laser modulation frequency *ω*_opt_ around $\omega_{1}\left(=\sqrt{\omega_{01}^{2}+K/2m}\right)$ , we make the system operate in the direct mode PiFM. The eigenmodes are expressed as linear combinations of *z*_1_ and *z*_2_, *Q*_1_ = −0.95*z*_1_ − *z*_2_, and *Q*_2_ = −1.05*z*_1_ + *z*_2_, and the motional shapes are depicted in Figure 2. Below each mode is the corresponding cantilever mode shape. The photo-induced force can be obtained by measuring the *Q*_1_ amplitude that is proportional to *F*_opt_, demodulated at *ω*_1_. The tip motion can be described by *z*_2_(*t*), which is a linear combination of *Q*_1_(*t*) and *Q*_2_(*t*) as ${\text{z}}_{2}={\text{U}}_{21}{\text{Q}}_{1}+{\text{U}}_{22}{\text{Q}}_{2}\approx -\frac{1}{2}\left(y_{1}+1\right){\text{Q}}_{1}-\frac{1}{2}\left(y_{1}-1\right){\text{Q}}_{2}$ = −0.525*Q*_1_ + 0.475*Q*_2_. The tip-sample distance is controlled by monitoring the second resonance frequency $\omega_{2}\approx \sqrt{\omega_{02}^{2}+K\left({\text{z}}_{0}\right)/2m}$ as a function of *z*_0_.

Next, we describe the SC mode. Since it involves nonlinear interaction such as frequency mixing, we should take one higher term in the Taylor expansion of *F*_opt_, which is *F*_opt_( $\left.{\text{z}}_{0}\right)-g{\text{z}}_{2}$ with *g* = d*F*_opt_/dz evaluated at *z* = *z*_0_.

$$\left(\begin{array}{c} {\ddot{Q}}_{1}\\ {\ddot{Q}}_{2} \end{array}\right)+\left(\begin{array}{cc} \gamma_{1} & 0\\ 0 & \gamma_{2} \end{array}\right)\left(\begin{array}{c} {\dot{Q}}_{1}\\ {\dot{Q}}_{2} \end{array}\right)+\frac{1}{m}\left(\begin{array}{cc} \lambda_{1} & 0\\ 0 & \lambda_{2} \end{array}\right)\left(\begin{array}{c} {\text{Q}}_{1}\\ {\text{Q}}_{2} \end{array}\right)$$

(10) $$\begin{array}{ll} \;\;\;\;\;\;\;\;\;\;\;\;\;\;\;\;\;\;\;\;\; & =\frac{1}{m}\left(\begin{array}{c} -\frac{1}{2}{\text{F}}_{\text{opt}}\left({\text{z}}_{0}\right)\text{cos}\left(\omega_{\text{opt}}\text{t}+\varphi \right)-{\text{F}}_{0}\text{cos}\omega_{\text{drv}}\text{t}\\ \frac{1}{2}{\text{F}}_{\text{opt}}\left(z_{0}\right)\text{cos}\left(\omega_{\text{opt}}\text{t}+\varphi \right)+{\text{F}}_{0}\text{cos}\omega_{\text{drv}}\text{t} \end{array}\right)\\ & \;+\frac{g}{m}\left(\begin{array}{c} z_{2}\text{cos}\left(\omega_{\text{opt}}\text{t}+\varphi \right)\\ -z_{2}\text{cos}\left(\omega_{\text{opt}}\text{t}+\varphi \right) \end{array}\right) \end{array}$$

Using the perturbation theory for *Q*_1_ = ${\text{Q}}_{1}^{\left(0\right)}+{\text{Q}}_{1}^{\left(1\right)}$ and ${\text{Q}}_{2}={\text{Q}}_{2}^{\left(0\right)}+{\text{Q}}_{2}^{\left(1\right)}$ , where ${\text{Q}}_{1}^{\left(0\right)}$ and ${\text{Q}}_{2}^{\left(0\right)}$ are the solutions for *g* = 0, we get the following equations.

(11) $$\begin{array}{ll} {\ddot{Q}}_{1}^{\left(1\right)}+\gamma_{1}{\dot{Q}}_{1}^{\left(1\right)}+\frac{\lambda_{1}}{m}Q_{1}^{\left(1\right)} & =\frac{g}{m}{\text{U}}_{21}Q_{1}^{\left(0\right)}\text{cos}\left(\omega_{\text{opt}}t+\varphi \right)\\ & \;+\frac{g}{m}U_{22}Q_{2}^{\left(0\right)}\cos\left(\omega_{\text{opt}}t+\varphi \right) \end{array}$$

(12) $$\begin{array}{ll} {\ddot{Q}}_{2}^{\left(1\right)}+\gamma_{2}{\dot{Q}}_{2}^{\left(1\right)}+\frac{\lambda_{2}}{m}Q_{2}^{\left(1\right)} & =-\frac{g}{m}{\text{U}}_{21}Q_{1}^{\left(0\right)}cos\left(\omega_{\text{opt}}t+\varphi \right)\\ & \;-\frac{g}{m}{\text{U}}_{22}Q_{2}^{\left(0\right)}\text{cos}\left(\omega_{\text{opt}}t+\varphi \right) \end{array}$$

Here, for the SC mode operation, the laser beam modulates at a frequency different from any of two resonance frequencies, *ω*_1_ or *ω*_2_, while the piezo-vibrator still oscillates at *ω*_drv_ = *ω*_2_. It means that the solution ${\text{Q}}_{1}^{\left(0\right)}$ of Eq. (6) can have two frequency components, *ω*_drv_ and *ω*_opt_ because neither of them is resonant with *ω*_1_. Therefore the driving terms of Eq. (11) have three frequencies, *ω*_opt_ ± *ω*_drv_, 2*ω*_opt_. Nevertheless, it can be easily found that the dominant contribution only comes from the second driving term. The solution is

$$Q_{1}^{\left(1\right)}\approx \frac{{\left[\frac{\text{d}{\text{F}}_{\text{opt}}}{\text{d}Z}\right]}_{0}{\text{U}}_{22}{\text{F}}_{0}}{2m^{2}\sqrt{{\left(\omega_{\text{s}+}^{2}-\omega_{01}^{2}-\frac{K}{2m}\right)}^{2}+\gamma_{1}^{2}\omega_{\text{s}+}^{2}}\sqrt{{\left(\omega_{\text{drv}}^{2}-\omega_{02}^{2}-\frac{K}{2m}\right)}^{2}+\gamma_{2}^{2}\omega_{\text{drv}}^{2}}}\text{cos}\left[\left(\omega_{\text{drv}}+\omega_{\text{opt}}\right)t+\delta +\varphi +\psi_{+}\right]$$

(13) $$\;\;\;\;\;\;\;\;\;\;\;\;\;\;\;\;\;\;\;\;+\frac{{\left[\frac{\text{d}{\text{F}}_{\text{opt}}}{\text{d}Z}\right]}_{0}{\text{U}}_{22}{\text{F}}_{0}}{2m^{2}\sqrt{{\left(\omega_{\text{s}-}^{2}-\omega_{01}^{2}-\frac{K}{2m}\right)}^{2}+\gamma_{1}^{2}\omega_{\text{s}-}^{2}}\sqrt{{\left(\omega_{\text{drv}}^{2}-\omega_{02}^{2}-\frac{K}{2m}\right)}^{2}+\gamma_{2}^{2}\omega_{\text{drv}}^{2}}}\text{cos}\left[\left(\omega_{\text{drv}}-\omega_{\text{opt}}\right)t+\delta -\varphi +\psi_{-}\right]$$

with *ω*_s+_ = *ω*_drv_ + *ω*_opt_, *ω*_s−_ = *ω*_drv_ − *ω*_opt_, *ψ*_+_ = $\text{ta}{\text{n}}^{-1}\frac{\gamma_{1}\omega_{\text{s}+}}{\omega_{\text{s}+}^{2}-{\left(\lambda_{1}/m\right)}^{2}}$ , and *ψ*_−_ = $\text{ta}{\text{n}}^{-1}\frac{\gamma_{1}\omega_{\text{s}-}}{\omega_{\text{s}-}^{2}-{\left(\lambda_{1}/m\right)}^{2}}$ .

The resonance condition for $Q_{1}^{\left(1\right)}$ is fulfilled by $\left|\omega_{\text{opt}}\pm \omega_{\text{drv}}\right|=\omega_{1}$ . It consequently results in double resonances because *ω*_drv_ is also close to *ω*_2_. For the solution $Q_{2}^{\left(1\right)}$ of Eq. (12), it cannot have resonance because *ω*_2_ is reserved for *ω*_drv_. The overall solutions for the SC mode are

$$Q_{1}=Q_{1}^{\left(0\right)}+Q_{1}^{\left(1\right)}\;$$

(14) $$\;\;\;\;\approx \frac{{\left[\frac{\text{d}{\text{F}}_{\text{opt}}}{\text{d}Z}\right]}_{0}{\text{U}}_{22}{\text{F}}_{0}}{2m^{2}\sqrt{{\left(\omega_{\text{s}-}^{2}-\omega_{01}^{2}-\frac{K}{2m}\right)}^{2}+\gamma_{1}^{2}\omega_{\text{s}-}^{2}}\sqrt{{\left(\omega_{\text{drv}}^{2}-\omega_{02}^{2}-\frac{K}{2m}\right)}^{2}+\gamma_{2}^{2}\omega_{\text{drv}}^{2}}}\text{cos}\left[\left(\omega_{\text{drv}}-\omega_{\text{opt}}\right)t+\delta -\varphi +\psi_{-}\right]$$

(15) $$Q_{2}={\text{Q}}_{2}^{\left(0\right)}+{\text{Q}}_{2}^{\left(1\right)}\approx \frac{{\text{F}}_{0}}{m\sqrt{{\left(\omega_{\text{drv}}^{2}-\omega_{02}^{2}-\frac{K}{2m}\right)}^{2}+\gamma_{2}^{2}\omega_{\text{drv}}^{2}}}\text{cos}\left(\omega_{\text{drv}}t+\delta \right)$$

where $Q_{1}^{\left(0\right)},{\text{Q}}_{2}^{\left(1\right)}$ , and the first term of Eq. (13) are nearly zero because those are all off-resonance. Notice that, while the signal of the direct mode is proportional to the force itself from Eq. (8), that of the SC mode is to the optical force gradient. By maintaining the *Q*_2_ mode amplitude constant to control the tip-sample gap, we can quantify the photo-induced force gradient through the observation of *Q*_1_ mode amplitude. We conclude that these solutions capture the principal features of PiFM, and are consistent with our previous results. In the following section, leveraging the results obtained thus far, we elucidate the distinctive signal behavior observed in the experimental outcome of a wedged polystyrene (PS) film. This explanation is achieved through a force model based on thermal expansion, followed by modulation of Van der Waals forces.

### 2.2 Thermal expansion force model: Van der Waals force modulation

PiFM was developed as a technique for investigating a sample’s optical properties by measuring the photo-induced force between the tip and the sample. Initially, this force was attributed to the attractive interaction between induced dipoles in the tip and the sample [14, 27, 28]. However, recent studies have suggested that in certain cases, light absorption followed by thermal expansion near the sample’s resonance wavelength can lead to changes in Van der Waals forces, resulting in a PiFM signal [29–31]. These thermal interaction forces typically dominate in polymers and organic materials with high optical absorption and thermal expansion coefficients. The spectral behavior of the thermal expansion force markedly differs from the interaction involving induced dipoles, which is primarily observed in materials with high oscillator strengths, such as metallic or polaritonic substances.

Thermal expansion force can be generated through two distinct mechanisms. One form of thermal expansion occurs over a larger area (∼a few µms) of the sample, illuminated by a far-field laser beam. This phenomenon, termed global expansion, occurs irrespective of the presence of the tip. Another form of expansion occurs in a much smaller region (∼a few nms) around the tip due to the absorption of the strong near-field generated around the sharp metallic tip. This phenomenon is referred to as tip-enhanced expansion and is responsible for the distinctive peak and valley shape observed in the PiFM signal plot as a function of sample thickness.

**Figure 3:**
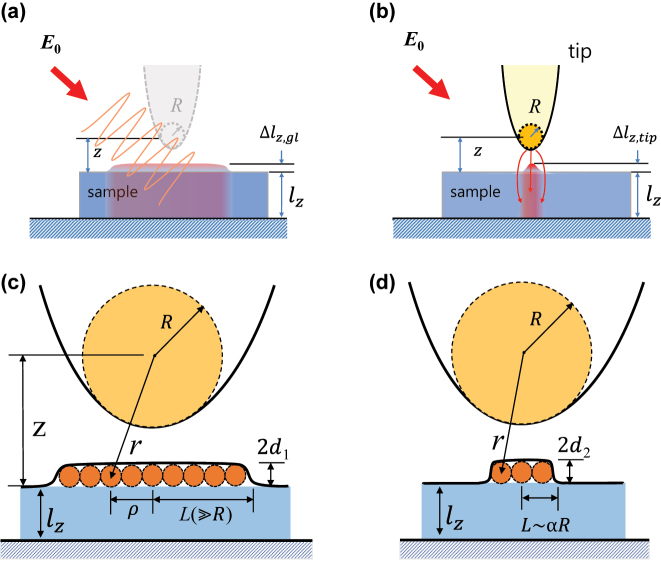
Schematic illustrations of (a) tip-enhanced thermal expansion and (b) global thermal expansion. (c) And (d) are simplified models for the corresponding thermal expansions, respectively. To apply the Van der Waals interaction between the tip and the sample, the tip is modeled as a large spherical body, and the expansion volume of materials as a collection of small spherical ones.

The thermal expansion force can be roughly modeled as shown in Figure 3. A sample of thickness *l*_*z*_, a low-index material such as polystyrene polymer, is deposited on a high-index substrate such as silicon or gold. Upon laser illumination at a resonance wavelength, the sample absorbs the radiation and heats up, leading to thermal expansion as illustrated in Figure 3a and b. The expansion corresponding to global thermal effects is denoted as Δ*l*_*z*,gl_, while that associated with tip-enhanced effects is represented as Δ*l*_*z*,tip_. Without the tip, it might be assumed that the laser field strength inside the sample is independent of the sample thickness, as there is nothing surrounding the sample to strongly influence the incident field. However, when the tip is present, the field strength becomes highly dependent on the sample thickness due to the near-field interaction between the metallic tip and the high-index substrate, which diminishes rapidly as the tip-substrate distance increases [29].

Here we approximate the tip as a large spherical entity and the expansion volume of materials as an aggregation of smaller spherical ones, as depicted in Figure 3c or d. Subsequently, we compute the interaction energy between the large sphere and the collection. By taking a derivative of this energy, we ultimately derive a force alteration that arises before and after expansion. This change in force can be regarded as the desired photo-induced force we seek to acquire. According to Ref. [32], the interaction energy between two spherical bodies with radii *R* and *d,* separated by a distance *r,* is expressed as

(16) $$\begin{array}{ll} U\left(r;R,d\right) & =-\frac{H_{\text{eff}}}{6}\left(\frac{2Rd}{r^{2}-{\left(R+d\right)}^{2}}+\frac{2Rd}{r^{2}-{\left(R-d\right)}^{2}}\right.\\ & \;+\left.\ln\left[\frac{r^{2}-{\left(R+d\right)}^{2}}{r^{2}-{\left(R-d\right)}^{2}}\right]\right) \end{array}$$

where *H*_eff_ is the effective Hamaker coefficient. For *R* ≫ *d*, it can be simplified by

(17) $$U\left(r;R,d\right)\approx -\frac{H_{\text{eff}}}{3}\frac{Rd}{r^{2}-R^{2}}$$

Assuming cylindrical symmetry around the tip axis, the expanded volume is thought to be a thin disk of thickness 2*d* and radius *L*. This approximation is reasonable because the volume of the spheres collection approaches that of the thin disk as the individual sphere size becomes very small. This holds true in our case where the thermal expansion thickness is far below one nm, very small compared to the tip diameter of 20 nm. The interaction energy between the tip and the disk is computed by integrating Eq. (17) over the entire disc area.

(18) $$\begin{array}{ll} U\left(z;R,L\right) & ∼-\frac{2\pi H_{\text{eff}}Rd}{3}\int_{0}^{L}\frac{1}{\rho^{2}+z^{2}-R^{2}}2\rho d\rho \\ & =-\frac{2\pi H_{\text{eff}}Rd}{3}\ln\left[\frac{z^{2}-R^{2}+L^{2}}{z^{2}-R^{2}}\right] \end{array}$$

For the global expansion *U*_gl_ (*L* ≫ *R*) and the tip-enhanced one *U*_tip_ (*L* = α*R*), we get, respectively

$${\text{U}}_{\text{gl}}\left(\text{z},\text{R},\text{L}\right)∼-\frac{2\pi {\text{H}}_{\text{eff}}\text{Rd}}{3}\text{ln}\left\{\frac{{\text{L}}^{2}}{{\text{z}}^{2}-{\text{R}}^{2}}\right\}$$

(19) $${\text{U}}_{\text{tip}}\left(\text{z},\text{R},\text{L}\right)∼-\frac{2\pi {\text{H}}_{\text{eff}}\text{Rd}}{3}\text{ln}\left[\frac{{\text{z}}^{2}-\left(1-\alpha^{2}\right){\text{R}}^{2}}{{\text{z}}^{2}-{\text{R}}^{2}}\right]$$

where *α* is a factor indicating how small the nearfield distribution concentrated near the tip is relative to the tip radius. The forces are, respectively

$${\text{F}}_{\text{gl}}∼-\frac{4\pi {\text{H}}_{\text{eff}}\text{Rd}}{3}\left[\frac{\text{z}}{{\text{z}}^{2}-{\text{R}}^{2}}\right]$$

(20) $${\text{F}}_{\text{tip}}∼-\frac{4\pi {\text{H}}_{\text{eff}}\text{Rd}}{3}\left[\frac{\alpha^{2}{\text{R}}^{2}\text{z}}{\left({\text{z}}^{2}-{\text{R}}^{2}\right)\left({\text{z}}^{2}-{\text{R}}^{2}+\alpha^{2}{\text{R}}^{2}\right)}\right]$$

The thermal expansion thickness 2*d* in Eq. (20) is expressed as below for global expansion 2*d*_1_ and tip-enhanced one 2*d*_2_, assuming 1-dimensional heat diffusion [31].

(21) $$\begin{array}{ll} 2d_{i} & =\sigma l_{z}\Delta T=\sigma l_{z}P_{i}\left(l_{z}\right)\frac{\tau_{\text{rel}}}{\rho C}\left[1-{\text{e}}^{-t_{p}/\tau_{\text{rel}}}\right]\;\text{with}\;\tau_{\text{rel}}\\ & =\frac{4}{\pi^{2}}\frac{\rho C}{\kappa_{\text{eff}}}l_{z}^{2} \end{array}$$

where *σ* is the thermal expansion coefficient, Δ*T* is the temperature increase, *t*_*p*_ is the laser illumination time, and *P*_*i*_ (*i* = 1,2) is the irradiation energy absorbed within the sample, *i* = 1 for global expansion case and *i* = 2 for tip-enhanced expansion case. And *ρ*, C, and *κ*_eff_ are the density, the heat capacity, and the effective thermal conductivity respectively.

Given that the absorbed laser energy is proportionate to the square of the electric field |*E*|^2^, the ratio *P*_2_/*P*_1_ equals to |*E*_tip_/*E*_inc_|^2^, where *E*_tip_ and *E*_inc_ represent the electric fields with and without the tip, respectively. As we just mentioned, the tip-enhanced field strength *E*_tip_ decreases as the sample thickness increases. This decrease is due to the effective electrostatic reflection factor, *β*_eff_ = (*ε*_eff_ − 1)/(*ε*_eff_ + 1), which diminishes with the increasing tip-substrate distance [29], where *ε*_eff_ is the effective permittivity of the sample including the substrate. Thus, it can be assumed that *E*_tip_ depends on the sample thickness *l*_*z*_ in an exponentially decreasing fashion as $E_{\text{tip}}=\left[q_{0}\right.\exp\left(-l_{z}/\delta ,\;\right.$ ) + B]*E*_inc_. The parameters *q*_0_, *δ*, and *B* are to be determined from experimental data. The inclusion of *B* accounts for the residual field that persists even at a very large sample thickness. Consequently, the absorbed energy *P*_2_ is strongly decaying with the sample thickness, while *P*_1_ remains constant depending only on the incident field *E*_inc_. In the following section, we will show the experimental results of sample thickness-dependent PiFM signals and explain why the signal plot in SC mode shows a peak followed by a valley through combining the models obtained so far.

## 3 Experimental methods and results

**Figure 4:**
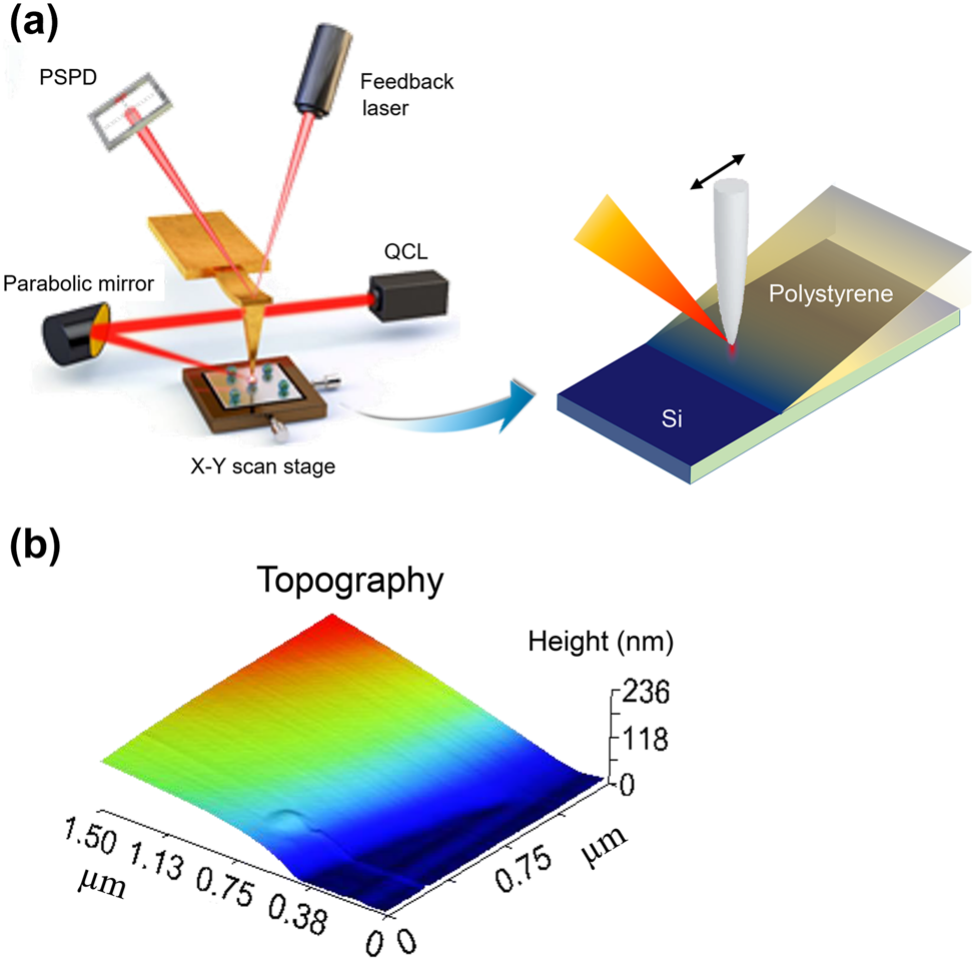
Schematic illustration of experimental apparatus. (a) Experimental setup of PiFM and illustration of the wedged PS sample under measurements. (b) Topography measurement of the sample, which was prepared simply by the drop-and-dry method.

The experimental setup is illustrated in Figure 4a. To observe the PiFM signal’s behavior as a function of sample thickness, a PS wedge sample was prepared by the drop-and-dry method of a homopolymer of PS onto a silicon substrate. The sample was placed on the microscope stage of a Vista-IR microscope from Molecular Vista Inc. and irradiated with a 50 ns mid-infrared pulsed laser beam at an angle of 40° from the sample surface by a parabolic mirror whose numerical aperture (NA) is around 0.4. The laser emission was at 1492 cm^−1^ from a Laser Tune QCL provided by Block Engineering. All measurements were conducted by gently tapping the sample with a gold-coated Si cantilevered tip, PPP-NCHAu, from Nanosensors Inc. (Switzerland). The tip diameter is about 20 nm. The fundamental resonance frequency is nominally 300 kHz and the second resonance is around 1.89 MHz. The pulsed QCL beam is modulated at a rate of the difference frequency of the cantilever’s first two eigenmodes at *f*_m_ = *f*_2_ − *f*_1_ = 1.59 MHz for the SC mode while it is at *f*_m_ = *f*_1_ = 300 kHz for the direct mode.

**Figure 5:**
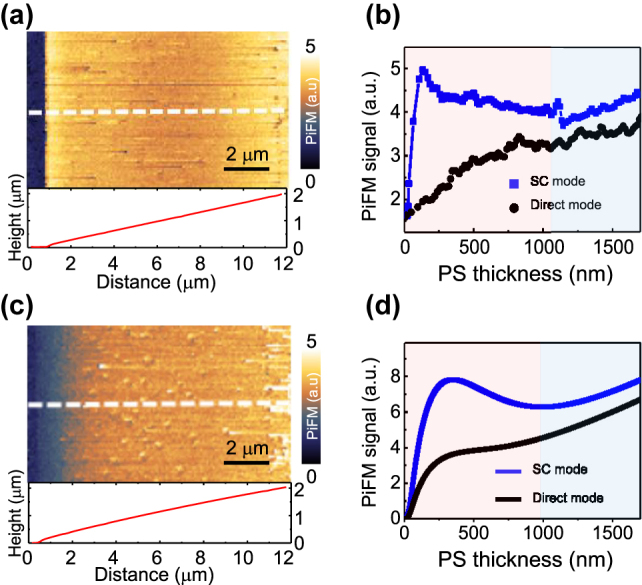
Experimentally measured PiFM images of the wedged PS sample in (a) SC mode and (c) direct mode, respectively. Below each image is the line profile of the corresponding topography. (b) Signal plots as functions of the PS thickness taken along the white dashed lines in the images, blue-filled squares for SC mode, and black-filled circles for direct mode, respectively. (d) Calculated PiFM signal plots as functions of the PS thickness, blue line for SC mode and black line for direct mode, respectively. The calculation model is based on the thermal expansion of the sample followed by Van der Waals force modulation that gives rise to the photo-induced force. The gradient of *F*_tip_ is dominant in the red-shaded region while that of *F*_gl_ is dominant in the blue-shaded region in SC mode.

As can be seen from the topography image in Figure 4b, the drop-and-dry method provides a fairly nice wedge shape as it moves inward from the drop edge. The PiFM images obtained from the two different operation modes are shown in Figure 5a and c, and two representative plots of the signal behaviors as functions of the sample thickness taken along the white dashed lines in the images are in Figure 5b. Manifestly the sideband coupling mode signals show a maximum followed by a minimum, then increase again with increasing thickness while in the direct mode the signals continuously increase.

As established in the coupled harmonic oscillators model in Section 2, the direct mode PiFM signal (Eq. (8)), *S*_d_, is proportional to the force acting on the tip, while the SC mode signal (Eq. (14)), *S*_sc_, is to the force gradient. Because two kinds of force simultaneously act on the tip, the signal of each mode is expressed by *S*_d_ ∼ *F*(*z*_0_) = *F*_gl_(*z*_0_) + *F*_tip_(*z*_0_) and *S*_sc_ ∼ [∂*F*/∂*z*]_*z*0_ = [∂*F*_gl_/∂*z*]_*z*0_ + [∂*F*_tip_/∂*z*]_*z*0_, where *F*_gl_ and *F*_tip_ are the forces due to global expansion and tip-enhanced expansion respectively. The force derivative with respect to *z* is the same as that with respect to *z*_2_. I.e. *z* − *R* = *z*_0_ − *z*_2_ from Section 2. Substituting Eq. (21) into Eq. (20) and forgetting all the common proportional constants, we get the force expressions as functions of sample thickness *l*_*z*_,

$${\text{F}}_{\text{gl}}\left({\text{l}}_{\text{z}}\right)∼{\text{l}}_{\text{z}}{\text{P}}_{1}\left({\text{l}}_{\text{z}}\right)\tau_{\text{rel}}\left[1-{\text{e}}^{-\frac{{\text{t}}_{\text{p}}}{\tau_{\text{rel}}}}\right]\left[\frac{\text{z}}{{\text{z}}^{2}-{\text{R}}^{2}}\right]$$

(22) $$\;\;\;\;\;\;\;\;\;\;\;\;\;\;\;={\text{P}}_{1}{\text{l}}_{\text{z}}^{3}\left[1-{\text{e}}^{-\frac{{\text{t}}_{\text{p}}}{\tau_{\text{rel}}}}\right]{\left[\frac{\text{z}}{{\text{z}}^{2}-{\text{R}}^{2}}\right]}_{{\text{z}}_{0}+\text{R}}$$

$$\begin{array}{ll} {\text{F}}_{\text{tip}}\left({\text{l}}_{\text{z}}\right) & ∼{\text{l}}_{\text{z}}{\text{P}}_{2}\left({\text{l}}_{\text{z}}\right)\tau_{\text{rel}}\left[1-{\text{e}}^{-\frac{{\text{t}}_{\text{p}}}{\tau_{\text{rel}}}}\right]\\ & \;\times \left[\frac{\alpha^{2}{\text{R}}^{2}\text{z}}{\left({\text{z}}^{2}-{\text{R}}^{2}\right)\left({\text{z}}^{2}-{\text{R}}^{2}+\alpha^{2}{\text{R}}^{2}\right)}\right] \end{array}$$

(23) $$\begin{array}{ll} \;\;\;\;\;\;\;\;\;\;\;\;\;\;\;\;\; & ={\text{q}}^{2}\left({\text{l}}_{\text{z}}\right){\text{P}}_{1}{\text{l}}_{\text{z}}^{3}\left[1-{\text{e}}^{-\frac{{\text{t}}_{\text{p}}}{\tau_{\text{rel}}}}\right]\\ & \;\times {\left[\frac{\alpha^{2}{\text{R}}^{2}\text{z}}{\left({\text{z}}^{2}-{\text{R}}^{2}\right)\left({\text{z}}^{2}-{\text{R}}^{2}+\alpha^{2}{\text{R}}^{2}\right)}\right]}_{{\text{z}}_{0}+\text{R}} \end{array}$$

where $q\left(l_{z}\right)=q_{0}\exp\left(-l_{z}/\delta ,\;\right.$ ) + *B*. Using Eqs. (22) and (23), we can calculate *S*_d_ and *S*_sc_ and plot them as in Figure 5d, where we use *R* = 10 nm, *t*_*p*_ = 50 ns, *ρ* = 1000 kg m^−3^, C = 1131 J kg^−1^ K^−1^, *κ*_eff_ = 0.038 W m^−1^ K^−1^, *α* = 0.1, *δ* = 500 nm, B = 1.2, *q*_0_ = 17.3. The tip-sample distance, *z* − *R*, is set 2 nm to correspond to the experimental condition. This value is sufficiently small to satisfy the requirement for the small oscillation approximation. The calculation from the models qualitatively explains well the experimental result. The observed peak and valley behavior in the plot can be attributed to the tip-enhancement effect arising from *β*_eff_, which diminishes as the tip moves away from the high-index substrate. And the effect is noticeable only in SC mode because the force drops faster with *z* in the tip-enhanced expansion case (∼1/*z* ^3^) than in global expansion one (∼1/*z*), provided that the force values are the same in both cases. The larger force gradient implies a dominant contribution to *S*_sc_. The signal eventually increases again over 1 μm because the global expansion force takes the job in a thick region, resulting in a peak followed by a valley even in a small oscillation limit, which cannot be well explained by the previous studies [29, 31]. The red-shaded regions in Figure 5b and d correspond to the small PS thickness range where the tip-enhanced field is strong enough to dominate the thermal expansion while the blue regions correspond to the global expansion dominance. In this region, field enhancement is hardly expected because the thick sample implies a large tip-sample distance.

**Figure 6:**
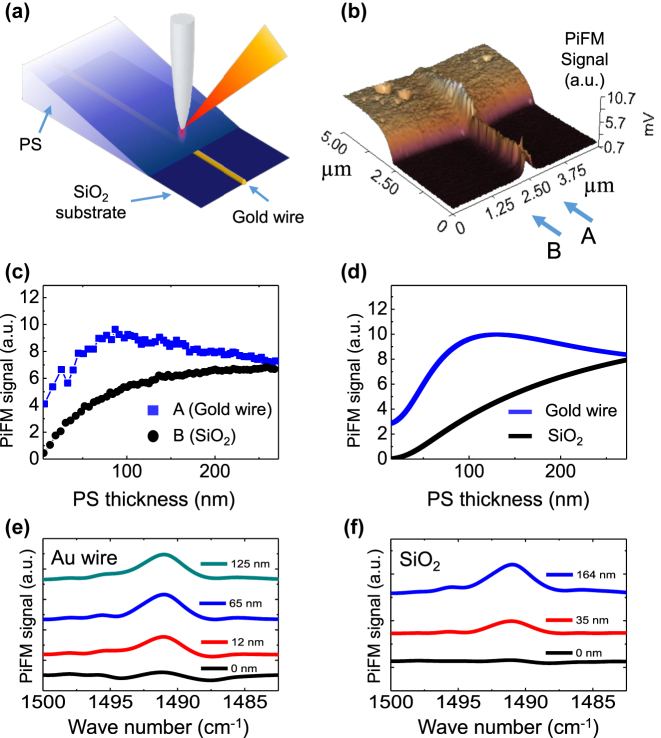
Experimental measurements and calculations of PiFM signals from a PS wedge. (a) Illustration of the wedged PS sample deposited on the low index SiO_2_ substrate having a gold nano-wire on top. (b) PiFM image measured in SC mode. (c) Signal plots as functions of the PS thickness along the directions designated by the two arrows in (b), blue-filled squares for the arrow A and black-filled circles for the arrow B, respectively. (d) Calculated PiFM signal plots as functions of the PS thickness on the gold wire (blue line) and on the SiO_2_ (black line), respectively. PiFM spectra on (e) gold wire and (f) SiO_2_ substrate.

Lastly, it is important to remark that this intriguing signal behavior could not be observed on low-index material substrates such as SiO_2_ because of low tip-enhancement. In order to illustrate this point, a PS wedge was deposited on a low-index SiO_2_ substrate with a gold nano-wire placed on top, as shown in Figure 6a. In Figure 6b and c are the SC mode PiFM image and two signal plots along the directions designated by two arrows. Clearly, the characteristic peak shape vanishes on the SiO_2_ substrate, despite being measured in SC mode. The signal exhibits a continuous increase with sample thickness. Interestingly, however, the signal on the gold wire shows the characteristic peak behavior because it can give rise to a high field enhancement effect between the tip and the metal wire. It can be readily found in Figure 6d that the calculation results with *q*_0_ = 9.5 and *δ* = 650 nm for the black curve and *q*_0_ = 22.3 and *δ* = 153 nm for the blue one, otherwise the same parameters as the Si substrate, correspond well with the experimental data qualitatively. This sample was not large enough to see a valley that would emerge at the thicker region. Due to the strong nearfield interaction between the tip and the bare gold wire, there is an appreciable amount of PiFM signal even at zero PS thickness. To account for this, a slight offset is applied to the blue plot in the calculations. Because the optical field is confined more tightly and stronger near metallic substance than semiconductors or organic insulators, the PiFM signal from the gold wire sample shows rapid growth, followed by a quick decrease, while maintaining a higher signal than the silicon-substrate sample or the glass-substrate one. This implies that the valley position may appear in thicker region compared to that of silicon substrate. Shown in Figure 6e and f are the PiFM spectra of PS measured at some representative heights of the wedge. The spectral response around the 1492 cm^−1^ infrared resonance of PS (polystyrene) also exhibits noticeable variations depending on the thickness in relation to the substrate materials. Figure 6e displays the SC mode PiFM spectra measured on an Au wire at different thickness positions: 0 nm, 12 nm, 65 nm, and 125 nm. It is evident that the IR resonance near 1492 cm^−1^ reaches its maximum at a thickness between 65 nm and 125 nm. However, the off-resonance spectra do not exhibit this behavior. Conversely, when we measure the SC mode PiFM spectra on a SiO_2_ substrate at thickness positions of 0 nm, 35 nm, and 164 nm, there is a gradual increase in the spectra as the thickness increases as in Figure 6f. These spectral responses clearly indicate that the observed thickness dependence is attributed to the thermal expansion of the PS rather than the induced dipole force.

## 4 Conclusions

In this paper, we present a coupled harmonic oscillators model comprised of two point masses connected by two massless elastic wires. This model aims to provide a simpler understanding of the operating principles of PiFM, making it easier to grasp key features of the technology in comparison to the cantilevered beam model. Solving the equations of motion with adjusted oscillator parameters, we could reproduce all the dynamics features in the beam model such as eigenmodes resonance frequencies and shapes, and motional responses to the various external forces in two different operation modes, direct and SC modes. Then we integrate this model with a thermal expansion force model to elucidate the intriguing signal behavior observed in SC PiFM mode, where the signal plot as a function of sample thickness unexpectedly shows a peak followed by a valley, which could not be well explained by the previous studies with small oscillation amplitude limit. Our findings indicate that this behavior stems from the tip-enhancement effect, influenced by the effective electrostatic reflection factor *β*_eff_ of the sample, which is decreasing as the tip goes away from the high-index substrate. The peak behavior is observed when the sample is on a metallic material, while it disappears on a low-index substrate. This observation underscores the pivotal role played by a high electrostatic reflection factor in the tip-enhancement effect. We conclude that, although our proposed model is currently qualitative, it effectively explains the fundamental physical mechanism underlying the intriguing PiFM signal behavior. It is anticipated that our model will facilitate a deeper understanding of various other physical phenomena associated with PiFM studies. We plan to apply this model to a hybrid PiFM-KPFM nanoscope system that utilizes three cantilever normal modes, enabling simultaneous measurements of photonic and electronic properties along with topography. In this case, particular attention will be given to the interplay between electric and optical forces under large oscillation amplitudes of the tip. By utilizing higher-order perturbation theory, which can be more easily developed in our new model than in the previous one, we aim to elucidate how the interaction between the two external forces affects the PiFM signal behavior.
